# Enrollment and Retention Outcomes from the Veterans Health Administration for a Remote Digital Health Study: Multisite Observational Study

**DOI:** 10.2196/68676

**Published:** 2025-08-15

**Authors:** Jaclyn A Pagliaro, Lauren K Wash, Ka Ly, Jenny Mathew, Alison Leibowitz, Ryan Cabrera, Jolie B Wormwood, Varsha G Vimalananda

**Affiliations:** 1 Center for Health Optimization and Implementation Research VA Bedford Medical Center Bedford, MA United States; 2 Neurological Clinical Research Institute Massachusetts General Hospital Boston, MA United States; 3 Clinical Informatics Providence VA Medical Center Providence, RI United States; 4 Aidar Health, Inc Columbia, MD United States; 5 Department of Psychology University of New Hampshire Durham, NC United States; 6 Chobanian & Avedisian School of Medicine Boston University Boston, MA United States

**Keywords:** enrollment, retention, clinical trial, remote digital health study, veterans, COVID-19

## Abstract

**Background:**

Clinical trials of remote patient monitoring (RPM) technology are well-suited to remote studies, for which patients complete key procedures online. However, remote digital health studies often suffer from low enrollment and retention, threatening the successful achievement of study outcomes and wasting resources and time. Recruiting patients from a large integrated health system offers a greater potential pool of participants for enrollment, which can increase the likelihood of successful study completion.

**Objective:**

This study describes enrollment and retention outcomes for a remote digital health study of an RPM device conducted in collaboration with researchers from the Veterans Health Administration (VA). The VA is the largest integrated health system in the United States, with 9 million enrollees who are, as a group, older and with more medical and mental health comorbidities than the civilian population.

**Methods:**

We aimed to enroll 200 VA patients for a clinical study of a cellular-enabled, handheld, multisensor device that captures multiple health parameters and transmits data to a cloud-based dashboard for viewing by clinicians. Eligible patients were hospitalized with COVID-19 within 3-6 months before enrollment and had one of 6 pre-existing medical comorbidities. Potentially eligible patients were identified using the VA Corporate Data Warehouse. Every 3 weeks, up to 1000 potentially eligible patients were mailed a recruitment letter. All study tasks, including obtaining informed consent, device training and troubleshooting, and handling study-related questions, were completed online and by telephone. Device and survey data were combined with VA clinical and utilization data to develop a predictive algorithm for clinical decompensation. The geographic distribution of enrolled patients was mapped by county. Demographic and health characteristics of nonenrolled versus enrolled, and of completers versus noncompleters were compared using *t* tests and chi-square tests as appropriate. Reasons for noncompletion were summed. Multivariate logistic regression was used to evaluate variables associated with enrolling versus nonenrolling, and completing versus noncompleting.

**Results:**

Of the 7714 who were mailed a study invitation, 560 were screened. Of the screened patients, 203 were enrolled (2.9% enrollment yield) and 166 completed the study (82% retention rate). Enrolled patients were broadly distributed across the United States. Among those enrolled, completers and noncompleters were similar except for a slightly higher proportion of patients with hypertension among completers. The most common reason for noncompletion of the study was that participants were unable to be contacted for study tasks.

**Conclusions:**

Remote digital health studies are increasingly common, but inadequate enrollment often results in failed studies. Recruiting patients through the VA enables access to a very large population of potentially eligible patients and can help ensure that clinical trials reach targets for enrollment and completion.

**Trial Registration:**

ClinicalTrials.gov NCT05713266; https://clinicaltrials.gov/study/NCT05713266

## Introduction

Digital health technologies such as remote patient monitoring (RPM) and mobile health apps enable health care to be provided outside the time and space of a clinical encounter, greatly expanding the potential reach of health care interventions. RPM can leverage wearables and connected medical equipment like weight scales and heart monitors to increase the volume, time period, and accessibility of health data available to clinicians for the management of chronic health conditions. Mobile apps may offer a range of tools, including education and counseling, diet and exercise tracking, and medication reminders. Since the nature of RPM and mobile apps is that individuals use them in their home environment, research to establish safety, efficacy, effectiveness, or validity can often be conducted remotely. Electronic data tools, including patient-facing technologies, can be used for consenting as well as intervention administration, remote interviews, outcome assessment, adherence monitoring, and data transmission [1,2]. Patients can thus participate from their own homes, with minimal or no travel to a study site. Studies of this type are variously described as remote, virtual, decentralized, direct-to-patient, and online. Daniore et al [3], define remote digital health studies as “longitudinal studies that use mobile technologies to conduct all key steps of a study completely online.”

Remote studies mimic the real-world use case for RPM and mobile apps. They also untether potential participants from the constraints of time and travel, which present known obstacles to sufficient enrollment [3]. A survey of cancer patients in the United States found that, if given the opportunity to enroll in a cancer clinical trial requiring travel further than their usual source of care, most respondents were more likely to participate if the trials used remote technology and decentralization tools to reduce the need for travel [4]. Yet remote digital health studies continue to face enrollment challenges, just as traditional digital and nondigital health studies do. These challenges can be difficult to overcome [5,6] and include lack of trust in clinical research, privacy concerns, participant time commitment, required effort, required experience with technology, and insufficient internal and external incentives [1,6-8]. Over 20% of clinical trials, including remote digital health studies, are terminated due to failure to meet enrollment targets [9]. One potential approach to meeting enrollment targets is to identify a large eligible sample at the outset, using large databases. This increases the likelihood that, even if the overall enrollment yield is low, enrollment targets will be met.

The Veterans Health Administration (VA), represents the nation’s largest integrated health system and offers a very large sample of patients from which to identify and recruit eligible participants. The VA includes over 9 million Veteran enrollees across the United States. VA provides health care at 170 VA Medical Centers and 1380 outpatient sites of care [10] that share a common underlying electronic health record with information rolled up into a national data warehouse. The data warehouse enables linkage of study data collected by RPM or mobile app data with a vast amount of VA clinical, health care usage, and cost data. VA-affiliated nonprofit centers offer flexibility for VA researchers to carry out studies with support from industry, foundations, academia, and other governmental agencies. Thus, the VA offers an ideal setting for large remote digital health studies.

In this paper, we report on study enrollment and completion among patients from the VA health system for a remote digital health study. The overall goal of the study was to develop an algorithm using data from a handheld RPM device to predict clinical decompensation (ie, worsening of existing symptoms or emergence of new symptoms). The study enrolled patients who were hospitalized with COVID-19 in the previous 3-6 months and the follow-up period was 6 months. Data collected from VA patients was provided to the industry sponsor for algorithm development. We describe the enrollment yield, characteristics of enrolled versus nonenrolled patients, and characteristics of patients who completed versus did not complete the study. Our results provide insights into the promise of successful remote digital health studies conducted in the VA health system and may help others plan similar studies in other large integrated health systems.

## Methods

### Setting

The VA health system serves over 7 million of its 9 million enrolled Veteran patients annually [10]. Veterans as a group are older than the civilian population, though women, who make up about 11% of VA users, tend to be younger. The largest age group of female VA patients is 35-44 years (22%), while the largest age group of male VA patients is 65-74 years (30%). Overall, 77% of VA patients are White, 18% of them are Black, and 8% of them are Hispanic. Veterans have a higher burden of physical and mental health comorbidities, including diabetes, obesity, smoking, depression, anxiety, and posttraumatic stress disorder [11-16]. Rates are also higher for substance use disorders, brain injuries, mental health disorders, and have a higher rate of committing suicide [17].

The health system includes 170 medical centers and nearly 1200 community-based outpatient clinics [10]. In many areas of the country, medical centers and clinics work together to offer inpatient, outpatient, and long-term care services in a geographic area. Across VA health care facilities, there is a single underlying platform for the electronic health record, which is called VistA. Clinicians conduct patient care using the VistA interface called the Computerized Patient Record System. Local instances of VistA are pushed into the national VA Corporate Data Warehouse (CDW) nightly. The CDW is a relational SQL server database that supports data analysis and reporting across multiple patients. The CDW can be queried to identify potentially eligible patients and also to gather longitudinal clinical and health care utilization data that can then be linked to data from digital health tools to examine outcomes of interest and their predictors.

### Study Design and Participants

This was an observational study of an RPM device. Details of the protocol, including information on recruitment procedures, have been published elsewhere [18]. The study team was based across 2 VA medical centers, with patients recruited from across the country without respect to their home VA facility. Briefly, Veterans were recruited to use a cellular-enabled, handheld, multisensory, FDA-approved device, MouthLab [19], that captures multiple health parameters through breath- and saliva-based biosensors and transmits the data to a cloud-based dashboard for viewing by clinical providers (ClinicalTrials.gov, NCT05713266). Enrolled patients used the device for 60 seconds twice daily over a 6-month follow-up period and filled out monthly web-based symptom surveys. Data from the device and surveys were combined with VA clinical and utilization data to support the development of a predictive algorithm for clinical decompensation among patients at high risk.

Patients aged 18 and older were eligible for the study if they had been hospitalized with a diagnosis of COVID-19 in the previous 3-6 months and had a pre-existing diagnosis of chronic obstructive pulmonary disease, asthma, hypertension, congestive heart failure, chronic kidney disease, or any type of diabetes mellitus as ascertained by CDW data using algorithms from the VA COVID-19 Shared Data Resource [20]. Access to as well as basic literacy with electronic platforms (ie, video calling and email) was an inclusion criterion. Patients with dementia were excluded given the coordinated physical effort required to use the device, and patients with pacemakers and implantable cardioverter-defibrillators were excluded due to the potential of these devices to interfere with device readings. Payments of US $85 were provided monthly for regular use of the device and completion of monthly web-based surveys. At the end of the study, return of the device qualified participants for up to US $175 additional, for a potential total payment of US $685.

The device developer in partnership with the Biomedical Advanced Research and Development Authority (BARDA), part of the US Department of Health and Human Services (HHS), funded the study, supplied devices to enrolled participants, supported training and device use, collected device data on a cloud-based platform, and developed the algorithm. The role of the VA team was to enroll patients, train them on the device, support device use throughout the study, collect monthly web-based survey data, and provide baseline as well as study period data on health conditions, medications, and health care usage. Health data was obtained from the VA CDW and via chart review of records obtained from non-VA care sources.

#### Recruitment

The study aimed to enroll 200 participants. All procedures were conducted remotely, with the principal investigator, the sponsor, and 5 study staff members spread across 5 different locations, sharing files via the VA-approved version of Box [21]. VA Box allows for subfolders that can be accessed by non-VA study staff outside the firewall. We used the CDW to identify potentially eligible Veterans across the United States and its territories. Every 3 weeks, up to 1000 potentially eligible patients were mailed a recruitment letter that summarized the study and provided the research team’s contact information.

#### Screening, Enrollment, and Device Training

Patients who called in response to the recruitment letter were provided basic information about the study and screened by telephone. If they were determined to be eligible, we scheduled a 30- to 45-minute video–based enrollment visit to thoroughly explain all study procedures and obtain informed consent. At the time of the enrollment visit, after study procedures were explained thoroughly, participants were emailed the informed consent and HIPAA (Health Insurance Portability and Accountability Act) authorization forms via the VA-approved version of DocuSign (Docusign, Inc). Patients had time to review those documents before choosing to electronically sign, which action automatically forwarded the forms to the study staff. The enrollment visit typically occurred on a day after the phone screening was completed. However, the visits could occur on the same day if the Veteran and study staff had time.

Following the study staff’s receipt of the signed informed consent, patients were considered enrolled. Enrolled patients were then called to schedule device training. Training was conducted over a 45- to 60-minute video visit after enrolled patients received shipment of their device directly from the sponsor. For all video visits, participants received an invitation to their email with the link to participate in the video visit using the VA-approved version of WebEx (Cisco), a videoconferencing platform.

Demographic information was available via the CDW for all potentially eligible patients. At the time of screening calls, patients volunteered their reasons for declining participation in their own words. Not all provided a reason for declining. Similarly, most but not all patients who withdrew provided a reason for doing so.

#### Analysis of Recruitment and Enrollment Data

Patient characteristics

Demographic characteristics used in the analysis were all from the CDW and included age, sex, marriage status, rurality, and race. VA priority group status was also included as a demographic characteristic. Priority groups are used to determine benefit eligibility. The highest priority group is Priority 1, 50%-100% disabled by a service-connected condition. Groups 2-4 capture lesser degrees of disability, as well as Purple Heart and Medal of Honor awardees, and former Prisoners of War. Priority group 5 is eligible on the basis of low income, and groups 6-8 have copays. Health conditions are identified as per the process for eligibility screening.

Analysis

Patient flow was described from mailing of the study invitation through study completion, or withdrawn, or dropped out status. Study completion was defined by patients having completed the final web-based survey. The geographic distribution of enrolled patients was mapped by county. Demographic characteristics and health conditions of nonenrolled versus enrolled patients, and of completers versus noncompleters, were compared using *t* tests and chi-square tests as appropriate. Multivariate logistic regression was used to evaluate variables associated with enrolling versus nonenrolling, and completing versus noncompleting. For both comparisons, all variables were entered simultaneously in a multivariable binary logistic regression predicting enrollment or completion status. Reasons for noncompletion were summed. Since reasons for declining or withdrawing were not always offered, and sometimes more than one reason was given, these data were presented descriptively without statistical analysis. We used SPSS (version 29.0.2; IBM Corp).

### Ethical Considerations

The protocol was approved by the Central institutional review board of the Veterans Healthcare Administration on December 21, 2022 (1720625-1). Since this was a multisite study, individual institutional review boards at the VA Bedford Healthcare System and the VA Providence Healthcare System also reviewed and approved the study. Informed consent was obtained from all eligible participants, after which HIPAA authorization was obtained. No study-related tasks were performed before obtaining both consent and HIPAA authorization. The informed consent form was sent to patients via DocuSign and reviewed with them on a WebEx video call. Participants receive the executed informed consent form via email. Participants received US$ 85 paid by check at the end of months 1-5, and upon completion of study tasks (survey and device return) in month 6, participants received US $175. The total potential compensation was US $600.

## Results

### Overview

**Figure 1:**
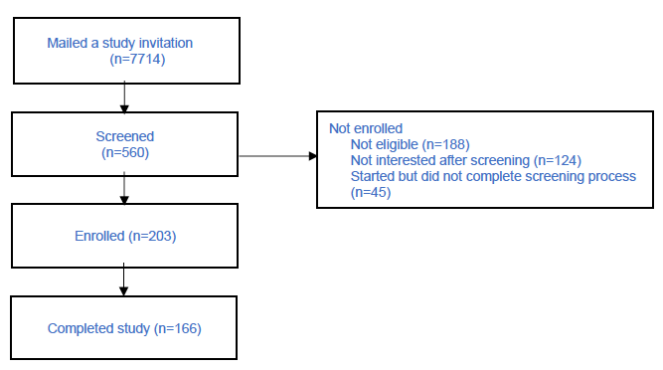
Flowchart of patient enrollment in a remote digital health observational study conducted in the Veterans Health Administration health care system.

**Figure 2:**
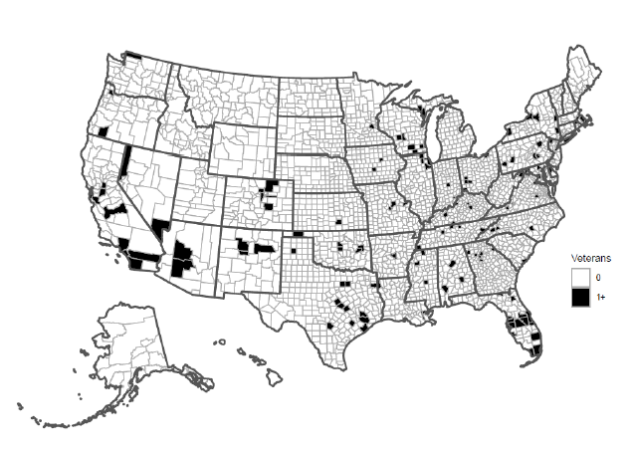
County-level distribution of enrolled participants in a remote digital health observational study conducted in the Veterans Health Administration health care system. Not pictured: participant locations in Puerto Rico (N=3).

Of the 7714 who were mailed a study invitation, 560 were screened (Figure 1). Of the screened patients, 203 were enrolled (2.6% enrollment yield) and 166 completed the study (82% retention rate). Enrolled patients were broadly distributed across the United States (Figure 2).

### Comparison of Patients Who Were Enrolled Versus Those Not Enrolled in the Study

Among those screened, characteristics of enrolled versus nonenrolled patients were very similar (Table 1). In bivariate analyses, enrolled patients were more likely to be married (55.7%) than unenrolled patients (40.3%; *P*<.001), and were more likely to have asthma (16.3%) than unenrolled patients (8.1%; *P*<.001). These differences were also significant in multivariable analyses controlling for all patient characteristics (Table 1). In addition, in multivariable analyses only, the odds of enrolling (vs not enrolling) were significantly higher among patients listed as Priority group 5 (eligible for VA benefits on the basis of low income; odds ratio 1.60, *P*=.02). No other differences were significant in bivariate or multivariable analyses.

**Table 1:** Comparing characteristics of eligible patients who did and those who did not enroll in a remote digital health observational study conducted in the Veterans Health Administration health care system.

Patient characteristics				Enrolled (N=203)	Not enrolled (N=7774)^a^	Bivariate *P* value	Multivariable^b^ (N=7337)	
							Odds ratio, 95% CI	*P* value
**Age**						.35^c^	0.88^d^ (0.75-1.04)	.13
			Years, mean (SD)	67.08 (10.26)	67.92 (12.53)			
**Sex (reference: female), n (%)**						.12	0.80 (0.46-1.36)	.41
		Female		19 (9.4)	513 (6.6)			
		Male		184 (90.6)	7261 (93.4)			.41
**Marriage status (reference: currently married), n (%)**						<.001^e^	2.12 (1.56-2.89)	<.001^e^
		Currently married		113 (55.7)	3136 (40.3)			
		Not currently married		90 (44.3)	4637 (59.6)			
		Missing		0 (0.0)	1 (0.0)		— ^f^	—
**Rurality (reference: not urban), n (%)**						.46	1.05 (0.64-1.70)	.86
		Urban		184 (90.6)	6915 (89)			.86
		Not urban		19 (9.4)	854 (11)			
		Missing		0 (0.0)	5 (0.1)		—	—
**Race (reference: not White), n (%)**						.71	0.89 (0.64-1.23)	.47
		White		127 (62.6)	4984 (64.1)			
		Not White		59 (29.1)	2183 (28.1)			
		Missing		17 (8.4)	607 (7.8)		—	—
**Priority status, n (%)**						.57		
		Group 1		72 (35.5)	3051 (39.2)		Reference	—
		Group 2, 3, or 4		40 (19.7)	1589 (20.4)		1.31 (0.87-1.96)	.20
		Group 5		61 (30)	2142 (27.6)		1.60 (1.09-2.34)	.02*
		Group 6, 7, or 8		30 (14.8)	973 (12.5)		1.36 (0.85-2.17)	.20
		Missing		0 (0.0)	19 (0.2)		—	—
**Comorbidities, n (%)**								
	Hypertension			183 (90.1)	6777 (87.2)	.21	1.48 (0.91-2.43)	.12
	Heart failure			41 (20.2)	1727 (22.2)	.504	0.96 (0.65-1.41)	.82
	Diabetes			89 (43.8)	3778 (48.6)	.18	0.91 (0.67-1.24)	.55
	Kidney disease			76 (37.4)	3419 (44)	.06	0.80 (0.58-1.09)	.16
	Asthma			33 (16.3)	631 (8.1)	<.001^e^	2.37 (1.57-3.57)	<.001^e^
	Chronic obstructive pulmonary disease			66 (32.5)	2478 (31.9)	.85	1.09 (0.79-1.51)	.60

^a^The total number of patients not enrolled was 7779, but 5 eligible individuals had no demographic or health data and so are excluded from analyses.

^b^All variables were entered simultaneously in a multivariable binary logistic regression predicting enrollment status.

^c^*P* value from an independent sample *t* test; all other bivariate *P* values from chi-square analyses. Missing data was omitted from all significance testing.

^d^Age was standardized before being entered into the multivariable model.

^e^Significant comparisons.

^f^Not available.

### Comparison of Patients Who Completed Versus Those Who Did Not Complete the Study

Among those enrolled, completers and noncompleters were also similar (Table 2). Those who completed the study were significantly more likely to have hypertension (92.2%) than those who did not complete the study (81.1%) in bivariate analyses (*P*=.04), though this difference was not significant in multivariable analyses controlling for all patient characteristics. No other differences were significant in bivariate or multivariable analyses (Table 2).

**Table 2:** Comparing characteristics of enrolled participants (N=203) who did and did not complete a remote digital health observational study conducted in the Veterans Health Administration health care system.

Patient characteristics				Completed (N=166)	Not Completed (N=37)	Bivariate *P* value	Multivariable ^a^ (N=186)	
							Odds ratio, 95% CI	*P* value
**Age**						.28^b^	1.34^c^ (0.78-2.31)	.29
			Years, mean (SD)	67.45 (9.83)	65.43 (12.04)			
**Sex (reference: female), n (%)**						.78	1.06 (0.23-4.91)	.94
	Female			16 (9.6)	3 (8.1)			
	Male			150 (90.4)	34 (91.9)			
**Marriage status (reference: currently married), n (%)**						.88	0.86 (0.38-1.95)	.72
		Currently married		92 (55.4)	21 (56.8)			
		Not currently married		74 (44.6)	16 (43.2)			
**Rurality (reference: not urban), n (%)**						.74	0.99 (0.29-3.46)	.99
		Urban		151 (91.0)	33 (89.2)			
		Not urban		15 (9.0)	4 (10.8)			
**Race (reference: not White), n (%)**						.93	0.86 (0.35-2.10)	.74
		White		104 (62.7)	23 (62.2)			
		Not White		48 (28.9)	11 (29.7)			
		Missing		14 (8.4)	3 (8.1)		— ^d^	—
**Priority status, n (%)**						.88		
		Group 1		58 (34.9)	14 (37.8)		Reference	—
		Group 2, 3, or 4		33 (19.9)	7 (18.9)		1 (0.35-2.89)	.10
		Group 5		49 (29.5)	12 (32.4)		0.92 (0.35-2.41)	.87
		Group 6, 7, or 8		26 (15.7)	4 (10.8)		1.55 (0.37-6.40)	.55
**Comorbidities, n (%)**								
	Hypertension			153 (92.2)	30 (81.1)	.04^e^	2.9 (0.88-9.58)	.08
	Congestive heart failure			33 (19.9)	8 (21.6)	.81	0.65 (0.23-1.84)	.42
	Diabetes			70 (42.2)	19 (51.4)	.31	0.75 (0.33-1.74)	.51
	Kidney disease			62 (37.3)	14 (37.8)	.96	0.9 (0.39-2.12)	.81
	Asthma			28 (16.9)	5 (13.5)	.62	1.59 (0.50-5.13)	.43
	Chronic obstructive pulmonary disease			55 (33.1)	11 (29.7)	.69	1.14 (0.45-2.88)	.78

^a^All variables entered simultaneously in a multivariable binary logistic regression predicting completion status. Missing data was omitted from all significance testing.

^b^*P* value from an independent samples *t* test; all other bivariate *P* values from chi-square analyses.

^c^Age was standardized before being entered into the multivariable model.

^d^Not applicable.

^e^Significant comparisons.

### Reasons for Noncompletion

The three most common reasons for noncompletion were that participants were unable to be contacted for study tasks (37.8% of noncompleters), difficulty with device functionality (21.6%), and health challenges making it difficult to adhere to the study protocol (13.5%). The 34 participants who dropped out or withdrew did so at a mean of 59.5 (SD 53.6) days and a median of 31 (IQR 30-72) days (Table 3).

**Table 3:** Reasons for noncompletion among enrollees in a remote digital health observational study conducted in the Veterans Health Administration health care system (N=37)a.

	n (%)
Unable to contact participant for necessary study tasks	14 (37.8)
Device functionality	8 (21.6)
Health challenges	5 (13.5)
Personal reasons	4 (10.8)
Deceased	2 (5.4)
5G coverage inadequate	2 (5.4)
Overcommitted	2 (5.4)
Not specified	3 (8.1)

^a^The total is greater than 37 because some participants had more than one reason for not completing the study.

## Discussion

### Principal Findings

Remote digital health studies are increasingly common, but challenges exist to adequate enrollment. While remote studies may support more equitable enrollment in terms of including patients who live at a distance from study sites, digital health studies using RPM or mobile apps often require access to broadband; sufficient digital health literacy to participate in online information sharing, consenting, training, and outcomes reporting; and ability to use the digital technology correctly. One approach to successful enrollment in the context of these challenges is to draw from a very large pool of potentially eligible patients. In this paper, we demonstrate how we accomplished this in the VA health system. Identification of potential participants using the VA national data warehouse, followed by a large-scale mailing effort, was a successful approach to meeting enrollment targets for our remote digital health study. In addition, we found minimal evidence of selection bias or attrition bias using our approach.

The value of access to a large cohort for recruitment for such studies is underscored by our overall recruitment yield of 2.6%. Low enrollment yield does raise concern for selection bias in the study. However, using available data to explore this possibility, we found minimal differences in the characteristics of those who enrolled and those who did not. Those enrolled were more likely to be married than those who did not enroll. One reason could be that, this study required the use of internet-based video and consent, and some patients required caregiver support to remember when and how to use the device. It is possible that those without social support from a spouse, some potential participants, were not able to manage the requirements of enrollment. We also found that low-income Veterans were represented more among enrollees than nonenrollees, likely reflecting the greater relative value of the participant payments to those in this group. It is possible that there were unmeasured differences in patients who enrolled and did not enroll, for example, related to digital health literacy or previous experience with the VA or with research. While these are concerns highly relevant to the equitable conduct of digital health studies in particular, such bias would not negatively impact the results of this particular study, which focused on associations between clinical parameters. Studies with different outcome measures may need to consider the potential for selection bias along other dimensions than those measured in this project.

Enrolled participants were widely dispersed across the United States. Interestingly, we did not enroll any participants from several states in the Midwest and Northeast, or Alaska, or Hawaii. This may be because these states tend to be more sparsely populated, so there were fewer potential participants in those states to start with. Others recruiting from VA may choose to oversample to increase the likelihood of recruitment from across states, or along any dimension that is of particular interest for the study, such as demographic characteristics, specific health conditions, or certain social determinants of health that are available in the national data.

The literature on demographic predictors of enrollment in digital health studies is relatively nascent [22], with inconsistent findings across studies [23]. For example, predictors of interest in one digital health study included clinical characteristics relevant to the study topic, but not age or sex [24]. Another study found that the odds of consent were lower for older and women patients [25]. Both studies were related to heart health.

Of those who enrolled, 82% completed the study. This is quite good, given that remote digital health studies have a median study completion of only 48% (IQR 35%-76%) [3]. Daniore et al [3], describe 3 criteria that impact the retention of participants in remote digital health studies. These are participant task complexity (frequency, complexity, and duration), participant motivation, including incentives and nudges (intrinsic and extrinsic), and scientific requirements (study design and sample size). We achieved a high completion rate despite a complex set of participant tasks: over a 6-month period, participants needed to attend a web-based training session that could last up to an hour, use the device twice a day, confirm data was uploaded each time, call and work with study staff for any needed device troubleshooting, and complete monthly web-based surveys. A counter to high task complexity was likely motivation. As noted above, participants expressed appreciation for the payments. In terms of intrinsic motivation, several participants expressed the desire to contribute to science, especially through research related to Veteran health and improving COVID-19 outcomes. This study did not provide clinical data from the device back to patients; doing so may have further strengthened extrinsic motivation to remain in the study. Interestingly, Daniore et al [3] found that interventional studies tend to have higher completion rates (median 55%, IQR 38%-79%) than do observational studies (median 43%, IQR 22%-60%). Although this study required that patients use the RPM device, we did not intervene in terms of changing any aspect of their health care. Nonetheless, we achieved a completion rate well above the median for either type of study.

It is helpful to consider ahead of time whether particular demographic groups may be more likely to drop out or withdraw from a study, so that the research team can develop plans to monitor and address differential retention. Other studies have found that lower age [5,26] and male as compared with female sex [26,27] predict dropout from digital health studies, which we did not observe in our study. Thoughtful and judicious use of digital technology for study tasks [28] may help boost retention across demographic groups. For digital health studies, as for traditional studies, sufficient numbers of specialized, persistent, and highly trained trial staff [28] with the ability to innovate and adapt strategies are particularly important [28,29], especially as the retention pattern specific to a given study may differ depending on how demographic characteristics interact with the 3 criteria of Daniore et al [3] described above.

### Limitations

Our study has limitations. In comparing characteristics of those who enrolled to those who did not, we were limited to those characteristics available in the CDW. Therefore, we were not able to examine the degree to which important factors like functional status, caregiver support, education, or digital literacy may have impacted enrollment. Those who had poor internet connectivity may have been more likely to not enroll or drop out, and those without internet connectivity could not participate in the study at all. We did not ask specifically why participants who did not complete the study dropped out or withdrew; we captured reasons that were offered spontaneously. It is thus possible that more detailed questioning would have revealed a richer understanding of reasons for noncompletion. For example, while several participants cited problems with the device as reasons for dropping out, problems such as slow data upload may actually have been related to cellular network capability in the participants’ location.

### Conclusions

Recruiting patients through the VA for remote digital health studies, using the CDW to identify potentially eligible patients, can help ensure that clinical trials reach targets for enrollment and completion. Collaboration with VA-affiliated researchers is required, which can be initiated either through direct contact with researchers, through the National Association of Veterans’ Research and Education Foundations [30], or through the VA Partnered Research Program [31]. Once a research collaboration is identified, local VA-affiliated nonprofit corporations provide a straightforward mechanism for collaboration with foundations, industry partners in pharmaceuticals and devices, universities, government agencies, and donors to advance research in VA.

## Abbreviations

BARDA: Biomedical Advanced Research and Development Authority
CDW: corporate data warehouse
HHS: Health and Human Services
HIPAA: Health Insurance Portability and Accountability Act
RPM: remote patient monitoring
VA: Veterans Health Administration

## Data Availability

The data that support the findings of this study are available from the authors on reasonable request.
